# Genetics: Polymorphisms, Epigenetics, and Something In Between

**DOI:** 10.1155/2012/867951

**Published:** 2011-11-29

**Authors:** Keith A. Maggert

**Affiliations:** Department of Biology, Texas A&M University, College Station, TX 77843, USA

## Abstract

At its broadest sense, to say that a phenotype is epigenetic suggests that it occurs without changes in DNA sequence, yet is heritable through cell division and occasionally from one organismal generation to the next. Since gene regulatory changes are oftentimes in response to environmental stimuli and may be retained in descendent cells, there is a growing expectation that one's experiences may have consequence for subsequent generations and thus impact evolution by decoupling a selectable phenotype from its underlying heritable genotype. But the risk of this overbroad use of “epigenetic” is a conflation of genuine cases of heritable non-sequence genetic information with trivial modes of gene regulation. A look at the term “epigenetic” and some problems with its increasing prevalence argues for a more reserved and precise set of defining characteristics. Additionally, questions arising about how we define the “sequence independence” aspect of epigenetic inheritance suggest a form of genome evolution resulting from induced polymorphisms at repeated loci (e.g., the rDNA or heterochromatin).

## 1. Epigenetics and Evolution

The importance of sequence polymorphisms in evolution is fundamental and irrefutable. The contribution of epigenetic gene regulation is considerably less well established. In this perspective, I will not attempt to summarize all the studies that have contributed to our current understanding of epigenetics; instead, I will thread together a handful of salient studies, taken particularly but not exclusively from Drosophila research, to illuminate how common and consequent “epigenetic” gene regulation may result from induced polymorphism. Inclusion of induced polymorphism in the panoply of epigenetic gene regulatory mechanisms may force us to reconsider our definitions, but is in accord with current and historic uses of “epigenetics,” and may provide a new mechanism to understand how stable changes in gene expression can be established and maintained.

**Figure 1:**
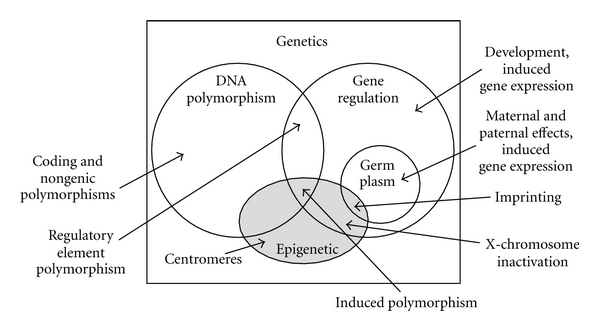
Relationships within genetics: random sequence polymorphisms, epigenetics, gene regulatory mechanisms, and induced polymorphisms.

To understand the role of epigenetics in evolution, it is necessary to consider definitions of both evolution and epigenetics. For the purpose of any discussion linking the two, “evolution” must expand to include the change of frequency of *phenotypic variants* irrespective of underlying allelic variants. This is a mild departure from a sequence-centric view of changes in allele frequencies in evolving populations, but is ironically more aligned with the original use of “epigenetic” to describe the abstract processes that produce a phenotype from a genotype prior to the elucidation of the central dogma, gene regulation, and developmental genetics. Now, “epigenetics” are instances of changes in gene regulation that do not correspond to underlying changes in nucleotide sequence. What one means by “changes in nucleotide sequence” is worth dwelling on, which I will do later. In general, changes in nucleotide sequence are “polymorphisms” although it is common to see them called “genetic” in order to contrast them with “epigenetic.” However, this is a misuse, and genetics is the study of inheritance and variation whatever their cause; polymorphism and epigenetics are subsets of genetics, and as I hope to convince you, they are neither exclusive nor exhaustive (Figure 1).

Understanding the joint contributions to evolution of polymorphism and epigenetics, particularly the latter, requires understanding the difference between them. This difference is profound since while polymorphisms are thought to be characterized by random, permanent, and well-understood changes to genetic information, epigenetic gene regulation is more volatile and hence has come to include induced and reversible alterations in heritable traits. This raises the popular view (however, unfair) of Jean-Baptiste de Lamarck, that our own actions or experiences may come to bear on our offspring. Lamarck envisioned that an organism evolves by passing its experience to offspring. On the surface, the inheritance of acquired characteristics was consistent with slow change in species over time. It was not until Weismann articulated the difficulty in a giraffe's neck discussing its experience with a giraffe's sperm that a Lamarckian mechanism of evolution was cast aside. The resurgence of Lamarckian models of evolution has recently occurred for a number of reasons. First, there are clear examples of inheritance of information outside of DNA sequence, which has opened the possibility of experience affecting gene expression and such changes in expression being transmitted to offspring. Second, not only is this hypothetical model possible, but it is heretical and provocative, and thus exciting. Third, perhaps many of us feel more than a little guilty in heaping ridicule on an otherwise superb scientist who happened to be wrong.

## 2. What Is/Are Epigenetics?

A clear, concise, and comprehensive definition of epigenetics is tricky to articulate, not because it is difficult *per se*, but because the term has seen an expansion over the last decade and has started to include things that are arguably not epigenetic. To clarify the situation, Youngson and Whitelaw gave a cogent description of the difference between two types of “epigenetics”: transmissible changes in expression (which they called “transgenerational epigenetic effects”) and transmissible chromosome modifications (“transgenerational epigenetic inheritance”) [1, 2]. They were attempting to separate two very different sets of phenomena that are both described as epigenetic. Many cases of “epigenetics” in recent literature fall into the former category and are not epigenetic at all, but rather are examples of germ cell gene regulation. To be meaningfully distinct from simple “transcription factor→enhancer→promoter→expression” forms of gene regulation, epigenetic phenomena must display three characteristics: they must manifest as (1) heritable genetic changes that (2) are associated with chromosomes but (3) are not based on DNA sequence. These are criteria that should not be abandoned, but should be evaluated.

The second characteristic is important because it is the essence of epigenetic inheritance. Why? Because if epigenetics did not require chromosome association, every genetic pathway that included a positive-feedback loop would be epigenetic. Female-specific sex-lethal splicing in Drosophila to form more active sex-lethal splicing factor would be considered epigenetic. Bacterial expression of LacY, the lactose permease, increasing sensitivity to further exposure to lactose would be considered epigenetic. Autophosphorylation of CaMKII upon witnessing a calcium spike would be considered epigenetic. Suppressor-of-Hairless-induced expression of Notch, the Suppressor-of-Hairless activator, would be considered epigenetic. In short, just about every genetic network could be considered epigenetic, and “epigenetics” would not differ in any meaningful way from “gene regulation.” Since proteins, lipids, RNAs, metabolic intermediates, and even toxins are passed through cell division in the cytoplasm, it is trivial to say that their effects are “inherited,” and it is wrong to conclude that cells retaining consequences of their antecedents' experiences are necessarily epigenetic.

Without requiring chromosomal inheritance of epigenetic phenomena, expression in the germ line would be sufficient to demarcate any genetic pathway as epigenetic, which would serve merely to rename those genes expressed during the creation of eggs and sperm. It should not be surprising that such networks might span multiple organismal generations; after all it is the mother's genetics (and experiences) that create the egg and the father's genetics (and experiences) that create the sperm; alteration in these processes will certainly result in alterations to the next generation. Mammalian biology aggravates the issue even more, since late-term pregnancies can involve three concentric organisms: by the end of gestation, female mammals contain half-genomes of all their potential grandchildren; the oocytes housing those pronuclei are filled with gene products created by their mothers from the nutritional environment provided by their grandmother. Many cases called epigenetic are instead this form of transgenerational gene regulation.

To discriminate broad concepts of “memory” or “potentiation” in gene regulation from specific epigenetic inheritance, it is necessary to show that the epigenetic factors altering gene activity map specifically to the chromosomal locus being regulated. Experimentally, *epigenetic gene regulation is demonstrated when DNA violates the law of mass action*: two identical sequences can act in different ways despite identity in their sequences and in the proteins that bind to them. Conceptually, if a “naive” DNA was introduced into the system, would it behave as do the existing DNAs? If so, it is not epigenetic. Practically, this is most easily shown by showing that identical pieces of DNA (homologs, duplications, transgenes, individuals of repeated gene arrays, etc.) possess different behaviors. This has been shown for centromere identity [3, 4], genomic imprinting in mammals [5], plants [6–8], fungi [9, 10], nematodes [11], and insects [12–14]; it is this requirement that many examples of “epigenetics” do not test. The strong connection between epigenetics and chromatin structure has only contributed to a conflation of these terms. It is not unusual to find the term “epigenetic” associated with studies that merely show changes in histone modifications of a gene, perhaps even acetylation, with no experiments that test for heritability, sequence polymorphism, or chromosome association.

## 3. Does Epigenetics Exist?

Changes in nucleotide sequence resulting in phenotypic variants are clear, established, and the very foundation of the neo-Darwinian synthesis that married Darwin's theories of variation and selection with Mendel's rules of inheritance. What was, and remains, magical about epigenetics is that substantial variation may be seen with no evident underlying changes in nucleic acid sequence and as such changes are relatively unstable. What first drew attention to epigenetic inheritance was the different behavior of identical genomes, in the variegation as a result of cosuppression which inactivates duplicated gene copies in plants, heterochromatin-induced position effects of Drosophila [15], or somatic mosaicism due to X chromosome inactivation in female mammals. These differences in phenotypes would not be surprising if they were due to differences in DNA sequence.

But how carefully have we tested for sequence identity in these cases? Imagine a hypothetical situation. What if creating a centromere required an enzyme (centromerase?) to cut the DNA and insert a specific sequence necessary and sufficient to establish centromere activity? What if cases of neocentromeres were cases of rare random expression and activity of centromerase? What if loss of centromeric activity in dicentric Robertsonian fusion chromosomes was evidence of the reversibility of centromerase? The hypothetical existence of centromerase is unnecessary, to be sure, given what we know about centromeric histones and chromatin structure, but it is illustrative that in many cases specific induced polymorphism is not even considered. We have a mindset that random mutation is the only mechanism allowed to alter DNA sequence, and therefore that rapid, induced, and reversible changes to chromosome behavior must occur without changes in sequence. But this assumes clearer lines in defining “sequence” than really exist, and it ignores many well-established observations.

Consider mating type switching in Schizosaccharomyces pombe. Switching occurs when a silent cassette of information from a “storage” locus is transferred to the active mating-type locus [16–18]. The mechanism of switching requires a mark, likely a break or ribonucleotide on one strand [19]. Tracing the ancestry of this strand has revealed that the altered strand comes from the switched locus in the previous generation. The result is that switching is limited in frequency and direction. A ribonucleotide in a chain of deoxyribonucleic acid is indeed a surprising way to carry information on a chromosome, but nonetheless it is genetic: it is heritable and consequent. And most surprising, it is inducible.

Consider also genomic imprinting in mammals. Is genomic imprinting really epigenetic? Although perhaps the most accepted form of epigenetics, it may be argued that it is not, for trivial nomenclatorial reasons: do you count 5-methylcytosine as cytosine, or as a fifth base that merely has an additional requirement for incorporation (a replication-coupled DNA methyltransferase)? While your answer may reveal something about your philosophy, it has impact on how we think of epigenetic mechanisms. If we count 5-methylcytosine as a fifth base, then the maternally and paternally derived alleles of genomically imprinted genes are indeed polymorphic. Can we also count dehydroxylation or deglycosylation as a polymorphism? Considering these cases of induced polymorphism would exclude both S. pombe mating type switching and imprinting at the Medea locus (where cytosine methylation induces a strand break on one homolog, alleviating it from silencing) as epigenetic. And why not? Selenocysteine is an amino acid even though a ribosome requires an extensive elaborated system to incorporate it [20, 21]. Methylcytosine is chemically and genetically distinct from cytosine; it merely requires an extensive elaborated system to incorporate it. A nicked DNA strand is again chemically and genetically distinct. It is a fun argument to make but seems overly contrived and unnecessary, and probably a little bizarre. It is not that we need to remove these cases from the list of epigenetics, but rather that we must consider what we mean by “sequence” when using it as the key criterion discriminating “epigenetics” from “polymorphisms.” There is a lot of landscape in that gray area.

## 4. Something In Between

Understanding how and why we define “sequence” and “epigenetic” is important when categorizing modes of gene regulation. But such considerations also reveal insight into how these phenomena might interact and lead us to consider how important induced polymorphism could be in evolution. The above examples—Medea, mating type, and imprinting—are all cases of induced polymorphism which result in changes in genetic activity of the sequence. The fact that they are “sequence independent” is an artifact of our ACGT-sequence bias. Still, it seems doubtful that these handful of examples would by themselves upset our views of evolution. First, such modes of epigenetic gene regulation are apparently uncommon. It is estimated that there are perhaps hundreds of imprinted loci in mammals, and as few as one in plants. Second, they are not cases of presence/absence of genetic pathways, but rather expression biases of different alleles, and so sibling do not differ markedly because of this mode of regulation; imprinted genes are essentially haploid and so are not much different than sex-linked genes in terms of evolution. Third, they are reset after one generation. It is therefore difficult to imagine that these forms of epigenetic inheritance drive evolution in profound or novel ways.

Are there examples of induced polymorphism that are widespread, consequent, and long-lived and might therefore affect genome evolution?

Almost one-half of the genomes of many popular metazoa are highly polymorphic, but those polymorphisms go unnoticed in genome-wide association, quantitative trait loci, and population genetics studies. This portion, the heterochromatin—alphoid and beta repeats, transposable elements, satellites, repetitive sequence, and so forth, all typically linked to centromeres—are not amenable to our modern approaches to genomics. Heterochromatin can comprise hundreds, thousands, and even millions of copies of simple (e.g., AATAT, AAGAG, and AAGAGAG) repeats [22–26]; hence they cannot be easily cloned, sequenced, or assembled using the techniques directed at whole-genome sequencing. In fact, the definition of “whole” has been altered to ignore this half of the genome [27–29]. Quantifying repeat copy number is cumbersome and imprecise, and stumbling upon rare sequence polymorphisms in otherwise homogenous blocks of satellite DNA is lucky [30]. It is therefore difficult to estimate the degree of differences or rate of polymorphism in this substantial portion of the genome.

Heterochromatin was first described by Emil Heitz in the 1920s and 1930s. At the time, its discriminating feature was heteropycnotic staining, which is still arguably the best definition. Subsequently, it was discovered that heterochromatin is generally late replicating, repressive for gene expression, and enriched in specific modifications of the DNA and the histones that package it although there are exceptions to all of these features [15, 31, 32]. What is agreed is that heterochromatin forms easily on highly-repetitive sequence and exists as a complex with heterochromatin proteins (e.g., histone methyltransferases, HP1, and possibly RNAs). Genetic and mutational manipulations that alter the amount of repetitive sequence or protein components demonstrate a natural balance between the sequence and protein components in forming heterochromatin [33–37]. Excess sequence compromises heterochromatin formation elsewhere by competing for limited heterochromatin proteins. Increases or decreases in heterochromatin proteins increase or decrease the ease of forming heterochromatin or increase or decrease the amount of sequence that can be packaged as heterochromatin.

Malik and Henikoff described their view of a specific example of an evolutionary balance at the centromeric chromatin (or “centrochromatin”) [38–40]. They envision a coevolution of sequence expansion and DNA binding by the centromeric histone Cid. Excess centromeric DNA is bound by Cid, and changes in Cid binding (or expression) result in altered centromeric sequence. This may be an example of a broader mechanism of expansion and contraction limited (or promulgated) by the characteristics of DNA-binding proteins that stabilize repetitious sequence. The mix of multiple polymorphic simple repeats in the genome [25, 26, 41, 42] may be stabilized by a mix of dedicated or overlapping heterochromatin proteins [43–48]. The balance between the sequences and proteins that together form heterochromatin is expected to be important because the protein components of heterochromatin play double duty as general transcriptional regulators [49, 50]. Genes shift between “heterochromatin-like” and “euchromatin-like” as they shift between silent and expressed during development or as a response to environmental stimuli. Mutations in the genes that encode these protein components often act dominantly, suggesting that their dose matters [34, 36]. One can easily imagine a three-way balance between heterochromatic sequence, heterochromatin proteins, and euchromatic gene regulatory mechanisms. This predicts that copy number polymorphisms of heterochromatin-forming sequence might impact gene regulation throughout the genome.

It has been very difficult to test whether copy number polymorphisms are consequential because there are few molecular-genetic tools that allow manipulation of copy number in otherwise isogenic backgrounds. We know from classic studies in Drosophila, where the DNA and protein components of heterochromatin are easily manipulated, that the amount of heterochromatic sequence in a cell dramatically affects sensitized variegating genes [33, 51, 52]. At an extreme, multiple supernumerary heterochromatic chromosomes are lethal [53]. Although the reason remains unclear, one can imagine such a disruption in sequence-to-protein balance to cause massive misregulation of many genes. Y chromosomes captured from wild populations vary in their ability to affect heterochromatin-induced position effect variegation and euchromatic gene expression elsewhere in the genome [54–56], yet have very few protein-encoding genes [57–59], strongly suggesting that heterochromatin polymorphisms, perhaps copy number polymorphisms, affect gene expression throughout the genome. Our work has induced copy number variation in one repeat, the ribosomal DNA (rDNA) [60]. The rDNA has precedent for housing-induced phenotypic variation in plants [61, 62], but without being able to induce changes at the rDNA, it had been difficult to test this phenomenon further. In flies, however, induced copy number variation has consequences for heterochromatin-induced position effect variegation and gene expression across the genome [63, 64]. These variations in gene regulation overlap with those seen from isolated natural Y chromosomes [54, 64], suggesting a significant portion of natural variance in rDNA repeat copy number [65, 66] may contribute to phenotypic variance in natural populations. Equally importantly, much of the variance that maps to the Y chromosome does not map to rDNA, suggesting that most phenotypic variance maps to other sequences on the Y, perhaps to the other repeats that are less experimentally manipulable.

Natural variation in repeat sequence copy number may play a role in evolution, but the uniquely dynamic biology of the rDNA implies the more exciting possibility. Changes in copy number may be induced and inherited.

The rDNA contains interspersed active and inactive rRNA genes and thus contains characteristics of both euchromatin and heterochromatin in some cells. The physical manifestation of the tremendous expression and processing of the rRNAs is the nucleolus. The stability of these long stretches of direct repeats in the nucleus is likely due to the heterochromatic packaging of a subset of the repeats. Peng and Karpen observed multiple nucleoli in postmitotic cells of animals carrying mutations of suppressor-of-variegation genes, which encode the protein components of heterochromatin and regulate the rDNA [67]. Their results suggest that repeat sequence not packaged as heterochromatin is unstable and prone to damage/repair or intrachromosomal recombination. Our experiments showed that mutation of suppressor-of-variegation genes resulted in destabilization and reduction of rDNA copy number through mitosis [63]. We further quantified loss in the soma and also showed that loss was seen through the germline, resulting in a permanent decrease of rDNA copy number in a population after exposure to a mutation that disrupts heterochromatin formation. We more recently showed that mutation of a repressor of rDNA expression (CCCTC-Factor, or CTCF) also destabilizes the rDNA, resulting in permanent loss [68]. These results are consistent with heterochromatin-like silencing stabilizing repeated DNA sequence, and a balance between repeat sequences and the protein components that regulate them.

In Drosophila, the ribosomal DNA is a compelling compartment because its dynamism is unmatched. It is the most highly expressed set of genes in the genome [69], coordinates the activity of all three polymerases, shrinks naturally through the formation of extrachromosomal circles [70], can repair itself through meiotic magnification or somatic pseudomagnification [71–75], and can compensate its output through alteration of elongation rate and possibly initiation rate [76, 77]. It possesses side-by-side copies that possess heterochromatic and euchromatic chromatin structures [76, 78–80]. As the central body in protein synthetic capacity, it is also responsive to nutritional status, sensitive to toxins and drugs, and susceptible to instability by alterations of gene products necessary for its regulation [81–87]. Altering regulation of the rDNA through mutation or drug treatment affects not only rRNA output, but also stability [88–90]. Alteration of the activity of a protein component of heterochromatin might therefore affect the copy number of the sequence to which it binds.

**Figure 2:**
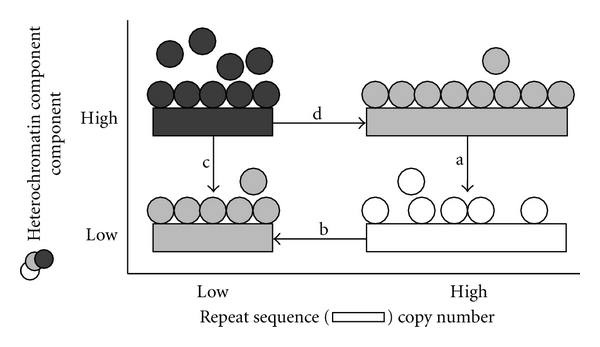
An illustration of a balance between heterochromatic sequences and heterochromatin components (e.g., proteins or RNAs). Repetitious heterochromatin-forming sequences (*rectangles*) are normally in balance with the proteins that bind them (*circles*), package them as heterochromatin, and thereby stabilize them (conditions in *gray*). Since these factors are used to regulate expression of euchromatic genes, the balance must accommodate “excess” factors for that purpose (denoted as circles apart from rectangles). If the expression or activity of proteins is reduced (a), repetitious sequence is exposed, destabilized, and lost through damage-repair, recombination, or extrachromosomal circle formation (b), until a new balance is established. Excess protein has gene regulatory consequence throughout the genome and presses to reestablish balance by altering expression level or activity (c) or perhaps through repeat expansion (d).

Dynamism (of rDNA) and balance (of heterochromatic sequence and proteins) establishes a situation of heterochromatin homeostasis (Figure 2). Sequences are protected from loss by packaging as heterochromatin. Loss of protein (or reduced protein activity, arrow “a”) would destabilize repeat DNA (white state) and result in loss, reestablishing an equilibrium (arrow “b” to the gray state). Similarly, excess sequence would revert through loss if there is not sufficient protein to package it for stability. But excess protein is not without consequence, since any heterochromatin protein not bound in constitutive sequence would alter gene expression throughout the genome (dark gray state), favoring either reduced protein expression/activity (arrow “c”) or expansion of repeat sequence (arrow “d”) to reestablish balance (light gray states). On the whole, the instability of repeat sequence and the consequence of excess heterochromatin proteins creates multiple states that balance the factors and naturally drives the number of repeat sequences and protein expression to equilibrate. Of course, any external factors that influence heterochromatin protein activity would be expected to result in induced and heritable changes in repetitive DNA copy number. The rDNA is particularly sensitive to induced copy number polymorphism, since it is affected by nutritional status throughout the lifetime of an organism and rDNA copy number exists in excess of what is required for translational demands, allowing some plasticity in copy number without being unduly disadvantageous.

 On the surface, induced copy number polymorphism is similar to epigenetic modification (particularly if one cannot easily sequence and assemble repetitious DNAs), and the ability of repeat sequences to change in copy number relatively easily adds the degree of volatility common in epigenetic gene regulation. Unlike many forms of transgenerational gene regulatory effects, induced copy number polymorphisms are linked to chromosomes, and thus are both heritable and selectable. Unlike epigenetic regulation of imprinted or inactivated chromosomes, induced copy number polymorphisms can be inherited over multiple generations. But like both transgenerational and epigenetic effects, the role of induced polymorphism is only beginning to be considered in evolution. Such investigation will likely be done in simple organisms, such as Drosophila, that have relatively simple rDNA architecture [91, 92]. By contrast, humans have multiple rDNA arrays which change in size frequently [93], and the complex regulation that renders some arrays active and others inactive means it may be some time before we understand how rDNA polymorphisms and rDNA instability [94] contribute to phenotypic variance in human population or to disease etiology.

## 5. Is the rDNA Special?

Induced polymorphism of rDNA copy number offers a convenient mechanism by which changes may be inherited although the same objections apply here as they do for the environmentally induced changes in gene expression that craned Lamarck's neck: how is the germline affected? In the case of induced polymorphism, germ cells may be more, not less, sensitive to induced alterations in heterochromatin composition, for three reasons. First, in many cases, gene expression is limited in these cell types. Perdurance of heterochromatin proteins, or the presence of ample gene product to endure fluctuation in gene activity, may be less in these cell types. Second, at least in males, the genome is stripped of most somatic chromatin components in favor of packaging proteins and polyamines. This may increase the sensitivity of such chromosomes to DNA rearrangements or specifically mark some regions for hypervariability. Third, germ cells naturally undergo recombination at a high rate. It is well established that changes in microsatellite and rDNA copy number occur in meiosis, while the same sequences are relatively stable in mitosis. The challenge is to understand what identifies a gene as “sensitive to rDNA copy number,” because it would be those genes selected for phenotypic variation in response to rDNA copy number changes.

We do not yet understand whether repeated sequences are different from “nonexpressed” sequences in ability to be induced to change, but we do know from mutational and molecular analyses that “heterochromatin” is not monolithic and is more accurately thought of as multiple “colors” [95]. Mutations may affect one chroma of heterochromatin and not another [96]. The five enumerated chromas significantly expand our understanding of chromatin structure, but even those five are likely still a simplification caused by our failure to resolve more subtle differences. Cumbersome work has detected alterations of repeat sequence copy number in few studies, suggesting that this may be a very widespread form of genetic variation [66, 97, 98]. Peng and Karpen showed an increase in DNA damage repair foci in the heterochromatin of suppressor-of-variegation mutants in diploid cells [99, 100]. They did not identify the sequences that were being repaired, but the number and distribution of repair foci in the nuclei indicated that it was not clustered (i.e., limited to the rDNA arrays). This observation suggests that the heterochromatin formed on simple repeats (and not just the highly-expressed rDNA) also is stabilized by packaging as heterochromatin. As our understanding of what heterochromatin is, and as tools become available to probe it in more surgical ways, we may begin to unravel complex interactions between types of heterochromatin as they struggle to keep each other in check or ally to fend off common enemies.

The term “epigenetics” may retain its strict definitions of chromosome-bound nonsequence-based genetic information, or it may be expanded to include induced mutation or gene regulatory networks that impact subsequent generations. In the end, all forms of regulation are genetic, and so are salient in understanding how complex, pleiotropic, and epistatic genetic interactions conspire to create phenotypes. However one defines epigenetics, it's legacy is that we cannot understand the comprehensive synthesis of forces that drive a genome's evolution without understanding how all the alleles within that genome are regulated.
